# Supplementary material for: Development and application of a NP-cELISA for the detection of nucleoprotein antibodies of equine influenza virus
Source: Microbiol Spectr. 2025 Aug 25;13(10):e00939-25. doi: 10.1128/spectrum.00939-25 (PMC12502703; doi:10.1128/spectrum.00939-25)
Supplement: Fig. S1 — Amino acids sequence alignment of NP from influenza types A (Equine, Chicken, Duck, Human Swine, Canine). [file spectrum.00939-25-s0001.pdf]

Alignment Report of Untitled.meg ClustalW (Slow/Accurate, Gonnet)

```
Majority                         M A S Q G T K R S Y E Q M E T G G E R Q N A T E I R A S V G R M V G G I G R F Y I Q M C T E L K L S
                                                  10                  20                  30                  40                  50

Equine-H3N8-EU794544.1.pro       M A S Q G T K R S Y E Q M E T D G E R Q N A T E I R A S V G R M V G G I G R F Y V Q M C T E L K L N  50
Equine-H3N8-KF806988.1.pro       . . . . . . . . . . . . . . . . . . . . . . . . . . . . . . . . . . . . . . . . . . . . . . . . . .  50
Equine-H3N8-M63786.1.pro         . . . . . . . . . . . . . . . G . . . . . . . . . . . . . . . . . . . . . . . . I . . . . . . . . G  50
Equine-H5N1-ON533586.1.pro       . . . . . . . . . . . . . . . G . . . . . . . . . . . . . . . . . S . . . . . . I . . . . . . . . S  50
Equine-H7N7-JX983551.1.pro       . . . . . . . . . . . . . . . G . . . . . . . . . . . . . . . . . . . . . . . . . . . . . . . . . S  50
Human-H1N1-CY034135.1.pro        . . . . . . . . . . . . . . . G . . . . D . . . . . . . . . . . I . . . . . . . I . . . . . . . . S  50
Human-H1N1-GQ166225.1.pro        . . . . . . . . . . . . . . . G . . . . D . . . . . . . . . . . I . . . . . . . I . . . . . . . . S  50
Human-H2N2-AY210104.1.pro        . . . . . . . . . . . . . . . . . . . . . . . . . . . . . . K . I D . . . . . . I . . . . . . . . S  50
Swine-H1N1-KP404381.1.pro        . . . . . . . . . . . . . . . G . . . . . . . . . . . . . . . . I . . . . . . . I . . . . . . Q . S  50
Chicken-H5N8-MF926454.1.pro      . . . . . . . . . . . . . . . G . . . . . . . . . . . . . . . . . . . . . . . . I . . . . . . . . S  50
Chicken-H7N7-KM593187.1.pro      . . . . . . . . . . . . . . . G . . . . . . . . . . . . . . . . . . . . . . . . I . . . . . . . . S  50
Chicken-H7N9-KM879323.1.pro      . . . . . . . . . . . . . . . G . . . . . . . . . . . . . . . . . S . . . . . . I . . . . . . . . S  50
Chicken-H9N2-AF156411.1.pro      . . . . . . . . . . . . . . . G . . . . . . . . . . . . . . . . . D . . . . . . I . . . . . . . . S  50
Duck-H5N1-AY856864.1.pro         . . . . . . . . . . . . . . . G . . . . . . . . . . . . . . . . . S . . . . . . I . . . . . . . . S  50
Duck-H5N2-JX507356.1.pro         . . . . . . . . . . . . . . . G . . . . . . . . . . . . . . . . . . . . . . . . I . . . . . . . . S  50
Duck-H9N2-KP865772.1.pro         . . . . . . . . . . . . . . . G . . . . . . . . . . . . . . . . . S . . . . . . I . . . . . . . . S  50
Canine-H3N2-MG254116.1.pro       . . . . . . . . . . . . . . . G . . . . D . . . . . . . . . . . I . . . . . . . I . . . . . . . . S  50
Canine-H5N2-HM765509.1.pro       . . . . . . . . . . . . . . . G . . . . . . . . . . . . . . . . . . . . . . . . I . . . . . . . . S  50

Majority                         D Y E G R L I Q N S I T I E R M V L S A F D E R R N K Y L E E H P S A G K D P K K T G G P I Y R R R
                                                  60                  70                  80                  90                 100

Equine-H3N8-EU794544.1.pro       D H E G R L I Q N S I T I E R M V L S A F D E R R N K Y L E E H P S A G K D P K K T G G P I Y R R K  100
Equine-H3N8-KF806988.1.pro       . . . . . . . . . . . . . . . . . . . . . . . . . . . . . . . . . . . . . . . . . . . . . . . . . .  100
Equine-H3N8-M63786.1.pro         . . . . . . . . . . . . . . . . . . . . . . . . . . . . . . . . . . . . . . . . . . . . . . . . . R  100
Equine-H5N1-ON533586.1.pro       . Y . . . . . . . . . . . . . . . . . . . . . . . . R . . . . . . . . . . . . . . . . . . . . . . R  100
Equine-H7N7-JX983551.1.pro       . . . . . . . . . . . . . . . . . . . . . . . . . . . . . . . . . . . . . . . . . . . . . . . . . R  100
Human-H1N1-CY034135.1.pro        . Y D . . . . . . . . . . . . . . . . . . . . . . . . . . . . . . . . . . . . . . . . . . . . . . V  100
Human-H1N1-GQ166225.1.pro        . Y D . . . . . . . . . . . . . . . . . . . . . . . . . . . . . . . . . . . . . . . . . . . . . . V  100
Human-H2N2-AY210104.1.pro        . Y . . . . . . . . L . . . . . . . . . . . . . . . . . . . . . . . . . . . . . . . . . . . . . . V  100
Swine-H1N1-KP404381.1.pro        . Y . . . . . . . . . . . . . . . . . . . . . . . . . . . . . . . . . . . . . . . . . . . . . K K R  100
Chicken-H5N8-MF926454.1.pro      . Y . . . . . . . . . . . . . . . . . . . . . . . . . . . . . . . . . . . . . . . . . . . . . . . R  100
Chicken-H7N7-KM593187.1.pro      . Y . . . . . . . . . . . . . . . . . . . . . . . . . . . . . . . . . . V . . . . . . . . . . . . . .  100
Chicken-H7N9-KM879323.1.pro      . N . . . . . . . . . . . . . . . . . . . . . . . . R . . . . . . . . . . . . . . . . . . . . . . R  100
Chicken-H9N2-AF156411.1.pro      . Y . . . . . . . . . . . . . . . . . . . . . . . . R . . . . . . . . . . . . . . . . . . . . . . R  100
Duck-H5N1-AY856864.1.pro         . Y . . . . . . . . . . . . . . . . . . . . . . . . R . . . . . . . . . . . . . . . . . . . . . . R  100
Duck-H5N2-JX507356.1.pro         . Y . . . . . . . . . . . . . . . . . . . . . . . . . . . . . . . . . . . . . . . . . . . . . . . R  100
Duck-H9N2-KP865772.1.pro         . N . . . . . . . . . . . . . . . . . . . . . . . . R . . . . . . . . . . . . . . . . . . . . . . R  100
Canine-H3N2-MG254116.1.pro       . Y . . . . . . . . . . . . . . . . . . . . . . . . . . . . . . . . . . . . . . . . . . . . . . . I  100
Canine-H5N2-HM765509.1.pro       . Y . . . . . . . . . . . . . . . . . . . . . . . . . . . . . . . . . . . . . . . . . . . . . . . R  100
```

```
Majority                       D G K W M R E L I L Y D K E E I R R I W R Q A N N G E D A T A G L T H L M I W H S N L N D A T Y Q R
                                                 110                           120                           130                           140                           150

Equine-H3N8-EU794544.1.pro     D G K W M R E L I L H D K E E I M R I W R Q A N N G E D A T A G L T H I M I W H S N L N D T T Y Q R  150
Equine-H3N8-KF806988.1.pro     . . . . . . . . . . Q . . . . . . . . . . . . . . . . . . . . . . . . . . . . . . . . . . . . . . .  150
Equine-H3N8-M63786.1.pro       . . . . . . . . . . Y . . . . . R . . . . . . . . . . . . . . . . . . L . . . . . . . . . A . . . .  150
Equine-H5N1-ON533586.1.pro     . . . . V . . . . . Y . . . . . R . . . . . . . . . . . . . . . . . . L . . . . . . . . . A . . . .  150
Equine-H7N7-JX983551.1.pro     . . . . . . . . . . . . . . . . . . . . . . . . . . . . . . . . . . . M . . . . . . . . . . . . . .  150
Human-H1N1-CY034135.1.pro      . . . . . . . . . . Y . . . . . R . V . . . . . . . . . . . . . . . . . . . . . . . . . . A . . . .  150
Human-H1N1-GQ166225.1.pro      . . . . . . . . . . Y . . . . . R . V . . . . . . . . . . . . . . . . . . . . . . . . . . A . . . .  150
Human-H2N2-AY210104.1.pro      . . . . . . . . V . Y . . . . . R . . . . . . . . . D . . . . . . . . M . . . . . . . . . . . . . .  150
Swine-H1N1-KP404381.1.pro      . . . . . . . . . . Y . . D . . R . . . . . . . . . . . . . . . . . . L . . . . . . . . . A . . . .  150
Chicken-H5N8-MF926454.1.pro    . . . . V . . . . . Y . . . . . R . . . . . . . . . . . . . . . . . . L . . . . . . . . . A . . . .  150
Chicken-H7N7-KM593187.1.pro    . . . . . . . . . . Y . . . . . R . . . . . . . . . . . . . . . . . . L . . . . . . . . . A . . . .  150
Chicken-H7N9-KM879323.1.pro    . . . . V . . . . . Y . . . . . R . . . . . . . . . . . . . . . . . . L . . . . . . . . . A . . . .  150
Chicken-H9N2-AF156411.1.pro    . . . . V . . . . . Y . . . . . R . . . . . . . . . . . . . . . . . . L . . . . . . . . . A . . . .  150
Duck-H5N1-AY856864.1.pro       . . . . V . . . . . Y . . . . . R . . . . . . . . . . . . . . . . . . L . . . . . . . . . A . . . .  150
Duck-H5N2-JX507356.1.pro       . . . . . . . . . . Y . . . . . R . . . . . . . . . . . . . . . . . . L . . . . . . . . . A . . . .  150
Duck-H9N2-KP865772.1.pro       . . . . V . . . . . Y . . . . . R . . . . . . Y . . . . . . . . . . . L . . . . . . . . . A . . . .  150
Canine-H3N2-MG254116.1.pro     . . . . . . . . . . Y . . . . . R . V . . . . . . . . . . . . . . . . . . . . . . . . . . A . . . .  150
Canine-H5N2-HM765509.1.pro     . . . . . . . . . . Y . . . . . R . . . . . . . . . . . . . . . . . . L . . . . . . . . . S . . . .  150

Majority                       T R A L V R T G M D P R M C S L M Q G S T L P R R S G A A G A A V K G V G T M V M E L I R M I K R G
                                                 160                           170                           180                           190                           200

Equine-H3N8-EU794544.1.pro     T R A L V R T G M D P R M C S L M Q G S T L P R R S G A A G A A V K G V G T M V M E L I R M I K R G  200
Equine-H3N8-KF806988.1.pro     . . . . . . . . . . . . . . . . . . . . . . . . . . . . . . . . . . . . . . . . . . . . . . . . . .  200
Equine-H3N8-M63786.1.pro       . . . . . . . . . . . . . . . . . . . . . . . . . . . . . . . . . . . . . . . . . . . . . . . . . .  200
Equine-H5N1-ON533586.1.pro     . . . . . . . . . . . . . . . . . . . . . . . . . . . . . . . . . . . . . . . . . . . . . . . . . .  200
Equine-H7N7-JX983551.1.pro     . . . . . . . . . . . . . . . . . . . . . . . . . . . . . . . . . . . . . . . . . . . . . . . . . .  200
Human-H1N1-CY034135.1.pro      . . . . . . . . . . . . . . . . . . . . . . . . . . . . . . . . . . . . . . I A . . . . . . . . . .  200
Human-H1N1-GQ166225.1.pro      . . . . . . . . . . . . . . . . . . . . . . . . . . . . . . . . . . . . . . I A . . . . . . . . . .  200
Human-H2N2-AY210104.1.pro      . . . . . . . . . . . . . . . . . . . . . . . . . . . . . . . . . . . . . . . . . . . . . . . . . .  200
Swine-H1N1-KP404381.1.pro      . . . . . . . . . . . . . . . . . . . . . . . . . . . . . . . . . . . . . . . . . . . . . . . . . .  200
Chicken-H5N8-MF926454.1.pro    . . . . . . . . . . . . . . . . . . . . . . . . . . . . . . . . . . . . . . . . . . . . . . . . . .  200
Chicken-H7N7-KM593187.1.pro    . . . . . . . . . . . . . . . . . . . . . . . . . . . . . . . . . . . . . . . . . . . . . . . . . .  200
Chicken-H7N9-KM879323.1.pro    . . . . . . . . . . . . . . . . . . . . . . . . . . . . . . . . . . . I . . . . . . . . . . . . . .  200
Chicken-H9N2-AF156411.1.pro    . . . . . . . . . . . . . . . . . . . . . . . . . . . . . . . . . . . . . . . . . . . . . . . . . .  200
Duck-H5N1-AY856864.1.pro       . . . . . . . . . . . . . . . . . . . . . . . . . . . . . . . . . . . . . . . . . . . . . . . . . .  200
Duck-H5N2-JX507356.1.pro       . . . . . . . . . . . . . . . . . . . . . . . . . . . . . . . . . . . . . . . . . . . . . . . . . .  200
Duck-H9N2-KP865772.1.pro       . . . . . . . . . . . . . . . . . . . . . . . . . . . . . . . . . . . I . . . . . . . . . . . . . .  200
Canine-H3N2-MG254116.1.pro     . . . . . . . . . . . . . . . . . . . . . . . . . . . . . . . . . . . I . . I A . . . . . . . . . .  200
Canine-H5N2-HM765509.1.pro     . . . . . . . . . . . . . . . . . . . . . . . . . . . . . . . . . . . . . . . . . . . . . . . . . .  200
```

```
Majority                         I N D R N F W R G E N G R R T R I A Y E R M C N I L K G K F Q T A A Q R A M M D Q V R E S R N P G N
                                                     210                 220                 230                 240                 250

Equine-H3N8-EU794544.1.pro       I N D R N F W R G E N G R R T R I A Y E R M C N I L K G K F Q T A A Q R A M M D Q V R E G R N P G N  250
Equine-H3N8-KF806988.1.pro       . . . . . . . . . . . . . . . . . . . . . . . . . . . . . . . . . . . . . . . . . . . . . . . . . .  250
Equine-H3N8-M63786.1.pro         . . . . . . . . . . . . . . . . . . . . . . . . . . . . . . . . . . . . . . . . . . . . S . . . . .  250
Equine-H5N1-ON533586.1.pro       . . . . . . . . . . . . . . . . . . . . . . . . . . . . . . . . . . . . . . . . . . . . S . . . . .  250
Equine-H7N7-JX983551.1.pro       . . . . . . . . . . . . . . . . . . . . . . . . . . . . . . . . . . . . . . . . . . . . . . . . . .  250
Human-H1N1-CY034135.1.pro        . . . . . . . . . . . . . . . . V . . . . . . . . . . . . . . . . . . . . . . . . . . . S . . . . .  250
Human-H1N1-GQ166225.1.pro        . . . . . . . . . . . . . . . . V . . . . . . . . . . . . . . . . . . . . . . . . . . . S . . . . .  250
Human-H2N2-AY210104.1.pro        . . . . . . . . . . . . . K . . S . . . . . . . . . . . . . . . . . . . . . . . . . . . S . . . . .  250
Swine-H1N1-KP404381.1.pro        . . . . . . . . . . . . . . . . . . . . . . . . . . . . . . . . . . . . . . . . . . . . S . . . . .  250
Chicken-H5N8-MF926454.1.pro      . . . . . . . . . . . . . . . . . . . . . . . . . . . . . . . . . . . . . . . . . . . . S . . . . .  250
Chicken-H7N7-KM593187.1.pro      . . . . . . . . . . . . . . . . . . . . . . . . . . . . . . . . . . . . . . . . . . . . S . . . . .  250
Chicken-H7N9-KM879323.1.pro      . . . . . . . . . . . . . . . . . . . . . . . . . . . . . . . . . . . . . . . . . . . . S . . . . .  250
Chicken-H9N2-AF156411.1.pro      . . . . . . . . . . . . . . P . . . . . . . . . . . . . . . . . . . . . . . . . . . . . S . . . . .  250
Duck-H5N1-AY856864.1.pro         . . . . . . . . . . . . . . . . . . . . . . . . . . . . . . . . . . . . . . . . . . . . S . . . . .  250
Duck-H5N2-JX507356.1.pro         . . . . . . . . . . . . . . . . . . . . . . . . . . . . . . . . . . . . . . . . . . . . S . . . . .  250
Duck-H9N2-KP865772.1.pro         . . . . . . . . . . . . . . . . . . . . . . . . . . . . . . . . . . . . . . . . . . . . S . . . . .  250
Canine-H3N2-MG254116.1.pro       . . . . . . . . . . . . . . . . . . . . . . . . . . . . . . . . . . . . . . . . . . . . S . . . . .  250
Canine-H5N2-HM765509.1.pro       . . . . . . . . . . . . . . . . . . . . . . . . . . . . . . . . . . . . . . . . . . . . S . . . . .  250

Majority                         A E I E D L I F L A R S A L I L R G S V A H K S C L P A C V Y G L A V A S G Y D F E R E G Y S L V G
                                                     260                 270                 280                 290                 300

Equine-H3N8-EU794544.1.pro       A E I E D L T F L A R S A L I L R G S V A H K S C L P A C V Y G L A V T S G Y D F E K E G Y S L V G  300
Equine-H3N8-KF806988.1.pro       . . . . . . . . . . . . . . . . . . . . . . . . . . . . . . . . . . . . . . . . . . . . . . . . . .  300
Equine-H3N8-M63786.1.pro         . . . . . . I . . . . . . . . . . . . . . . . . . . . . . . . . . . . A . . . . . . R . . . . . . .  300
Equine-H5N1-ON533586.1.pro       . . . . . . I . . . . . . . . . . . . . . . . . . . . . . . . . . . . A . . . . . . R . . . . . . .  300
Equine-H7N7-JX983551.1.pro       . . . . . . I . . . . . . . . . . . . . . . . . . . . . . . . . . . . A . . . . . . . . . . . . . .  300
Human-H1N1-CY034135.1.pro        . . . . . . I . . . . . . . . . . . . . . . . . . . . . . . . . . . . A . . H . . . R . . . . . . .  300
Human-H1N1-GQ166225.1.pro        . . . . . . I . . . . . . . . . . . . . . . . . . . . . . . . . . . . A . . H . . . R . . . . . . .  300
Human-H2N2-AY210104.1.pro        . . . . . . I . . . . . . . . . . . . . . . . . . . . . . . . . P . . A . . . . . . . . . . . . . .  300
Swine-H1N1-KP404381.1.pro        . . . . . . I . . . . . . . . . . . . . . . . . . . . . . . . . . V . A . . . . . . R . . . . . . .  300
Chicken-H5N8-MF926454.1.pro      . . . . . . I . . . . . . . . . . . . . . . . . . . . . . . . . . . . A . . . . . . R . . . . . . .  300
Chicken-H7N7-KM593187.1.pro      . . . . . . I . . . . . . . . . . . . . . . . . . . . . . . . . . . . A . . . . . . R . . . . . . .  300
Chicken-H7N9-KM879323.1.pro      . . . . . . I . . . . . . . . . . . . . . . . . . . . . . . . . . . . A . . . . . . R . . . . . . .  300
Chicken-H9N2-AF156411.1.pro      V . . . . . I . . . . . . . . . . . . . . . . . . . . . . . . . . . . A . . . . . . R . . . . . . .  300
Duck-H5N1-AY856864.1.pro         . . . . . . I . . . . . . . . . . . . . . . . . . . . . . . . . . . . A . . . . . . R . . . . . . .  300
Duck-H5N2-JX507356.1.pro         . . . . . . I . . . . . . . . . . . . . . . . . . . . . . . . . . . . A . . . . . . R . . . . . . .  300
Duck-H9N2-KP865772.1.pro         . . . . . . I . . . . . . . . . . . . . . . . . . . . . . . . . . . . A . . . . . . R . . . . . . .  300
Canine-H3N2-MG254116.1.pro       . . . . . . I . . . . . . . . . . . . . . . . . . . . . . . . . . . . A . . H . . . R . . . . . . .  300
Canine-H5N2-HM765509.1.pro       . . . . . . I . . . . . . . . . . . . . . . . . . . . . . . . . . . . A . . . . . . R . . . . . . .  300
```

```
Majority                       I D P F R L L Q N S Q V F S L I R P N E N P A H K S Q L V W M A C H S A A F E D L R V S S F I R G T
                                                 310                 320                 330                 340                 350

Equine-H3N8-EU794544.1.pro     I D P F K L L Q N S Q I F S L I R P K E N P A H K S Q L V W M A C H S A A F E D L R V L N F I R G T  350
Equine-H3N8-KF806988.1.pro     . . . . . . . . . . . . . . . . . . . . . . . . . . . . . . . . . . . . . . . . . . . . . . . . . .  350
Equine-H3N8-M63786.1.pro       . . . . R . . . . . . V . . . . . . N . . . . . . . . . . . . . . . . . . . . . . . . S S . . . . .  350
Equine-H5N1-ON533586.1.pro     . . . . R . . . . . . V . . . . . . N . . . . . . . . . . . . . . . . . . . . . . . . S S . . . . .  350
Equine-H7N7-JX983551.1.pro     . . . . . . . . . . . . . . . . . . . . . . . . . . . . . . . . . . . . . . . . . . . S . . . . . .  350
Human-H1N1-CY034135.1.pro      . . . . . . . . . . . V V . . M . . N . . . . . . . . . . . . . . . . . . . . . . . . S S . . . . K  350
Human-H1N1-GQ166225.1.pro      . . . . . . . . . . . V V . . M . . N . . . . . . . . . . . . . . . . . . . . . . . . S S . . . . K  350
Human-H2N2-AY210104.1.pro      . . . . . . . . . . . V Y . . . . . N . . . . . . . . . . . . . . N . . . . . . . . . . S . . . . .  350
Swine-H1N1-KP404381.1.pro      . . . . R . . . S . . V . . . . . . N . . . V . . . . . I . . . . . . . . . . . . . . S S . . . . .  350
Chicken-H5N8-MF926454.1.pro    . . . . R . . . . . . V . . . . . . N . . . . . . . . . . . . . . . . . . . . . . . . S S . . . . .  350
Chicken-H7N7-KM593187.1.pro    . . . . R . . . . . . V . . . . . . N . . . . . . . . . . . . . . . . . . . . . . . . S S . . . . .  350
Chicken-H7N9-KM879323.1.pro    . . . . R . . . . . . V . . . . . . N . . . . . . . . . . . . . . . . . . . . . . . . S S . . . . .  350
Chicken-H9N2-AF156411.1.pro    . . . . R . . . . . . V . . . . . . N . . . . . . . . . . . . . . . . . . . . . . . . S S . . . . .  350
Duck-H5N1-AY856864.1.pro       . . . . R . . . . . . V . . . . . . N . . . . . . . . . . . . . . . . . . . . . . . . S S . . . . .  350
Duck-H5N2-JX507356.1.pro       . . . . R . . . . . . V . . . . . . N . . . . . . . . . . . . . . . . . . . . . . . . S S . . . . .  350
Duck-H9N2-KP865772.1.pro       . . . . R . . . . . . V . . . . . . N . . . . . . . . . . . . . . . . . . . . . . . . S S . . . . .  350
Canine-H3N2-MG254116.1.pro     . . . . . . . . . . . V V . . M . . N . . . . . . . . . . . . . . . . . . . . . . . . S S . . . . K  350
Canine-H5N2-HM765509.1.pro     . . . . R . . . . . . V . . . . . . N . . . . . . . . . . . . . R . . . . . . . . . . S S . . . . .  350

Majority                       R V V P R G Q L S T R G V Q I A S N E N M E T M D S S T L E L R S R Y W A I R T R S G G N T N Q Q R
                                                 360                 370                 380                 390                 400

Equine-H3N8-EU794544.1.pro     K V I P R G Q L A T R G V Q I A S N E N M E T I D S S T L E L R S K Y W A I R T R S G G N T S Q Q R  400
Equine-H3N8-KF806988.1.pro     . . . . . . . . . . . . . . . . . . . . . . . . . . . . . . . . . R . . . . . . . . . . . . N . . .  400
Equine-H3N8-M63786.1.pro       R M V . . . K . S . . . . . . . . . . . . . . M . . N . . . . . . . . . . . . . . . . . . . N K . .  400
Equine-H5N1-ON533586.1.pro     R . V . . . . . S . . . . . . . . . . . . G A M . . N . . . . . . R . . . . . . . . . . . . N . . .  400
Equine-H7N7-JX983551.1.pro     . . . . . . . . . . . . . . . . . . . . . . . . . . . . . . . . . R . . . . . . . . . . . . . . . .  400
Human-H1N1-CY034135.1.pro      . . . . . . K . S . . . . . . . . . . . V . . M . . N . . . . . . R . . . . . . . . . . . . N . . K  400
Human-H1N1-GQ166225.1.pro      . . . . . . K . S . . . . . . . . . . . V . . M . . N . . . . . . R . . . . . . . . . . . . N . . K  400
Human-H2N2-AY210104.1.pro      . . S . . . K . S . . . . . . . . . . . . D . M G . . . . . . . . R . . . . . . . . . . . . N . . .  400
Swine-H1N1-KP404381.1.pro      . . V . . . . . S . . . . . . . . . . . . . . M . . I . . . . . . . . . . . . . . . . . . . N . . .  400
Chicken-H5N8-MF926454.1.pro    R . V . . . . . S . . . . . . . . . . . . . . M . . . . . . . . . R . . . . . . . . . . . . N . . .  400
Chicken-H7N7-KM593187.1.pro    R . V . . . . . S . . . . . . . . . . . . . . M . . . . . . . . . R . . . . . . . . . . . . N . . .  400
Chicken-H7N9-KM879323.1.pro    R M V . . . . . S . . . . . . . . . . . . . A M . . N . . . . . . R . . . . . . . . . . . . N . . .  400
Chicken-H9N2-AF156411.1.pro    R . V . . . . . S . . . . . . . . . . . . . . M . . . . . . . . . R . . . . . . . . . . . . N . . .  400
Duck-H5N1-AY856864.1.pro       R . V . . . . . S . . . . . . . . . . . . . A M . . N . . . . . . R . . . . . . . . . . . . N . R .  400
Duck-H5N2-JX507356.1.pro       R . V . . . . . S . . . . . . . . . . . . . . M . . . . . . . . . R . . . . . . . . . . . . N . . .  400
Duck-H9N2-KP865772.1.pro       R M V . . . . . S . . . . . . . . . . . I . A M . . N . . . . . . R . . . . . . . . . . . . N . . .  400
Canine-H3N2-MG254116.1.pro     . . . . . . K . S . . . . . . . . . . . V . . M . . N . . . . . . R . . . . . . . . . . . . N . . K  400
Canine-H5N2-HM765509.1.pro     R . V . . . . . S . . . . . . . . . . . . . . M . . . . . . . . . R . . . . . . . . . . . . N . . .  400
```

```
Majority                     A S A G Q I S V Q P T F S V Q R N L P F E R A T I M A A F T G N T E G R T S D M R T E I I R M M E S
                                              410                 420                 430                 440                 450

Equine-H3N8-EU794544.1.pro   A S A G Q I S V Q P T F S V Q R N L P F E R A T I M A A F T G N T E G R T S D M R T E I I R M M E S  450
Equine-H3N8-KF806988.1.pro   . . . . . . . . . . . . . . . . . . . . . . . . . . . . . . . . . . . . . . . . . . . . . . . . . .  450
Equine-H3N8-M63786.1.pro     . . . . . . . . . . . . . . . . . . . . . . . . . . . . . . . . . . . . . . . . . . . . . . . . . .  450
Equine-H5N1-ON533586.1.pro   . . . . . . . . . . . . . . . . . . . . . . . . . . . . . . . . . . . . . . . . . . . . . . . . . .  450
Equine-H7N7-JX983551.1.pro   . . . . . . . . . . . . . . . . . . . . . . . . . . . . . . . . . . . . . . . . . . . . . . . . . N  450
Human-H1N1-CY034135.1.pro    . . . . . . . . . . . . . . . . . . . . . . . . V . . . . S . . N . . . . . . . . . . V . . . . . .  450
Human-H1N1-GQ166225.1.pro    . . . . . . . . . . . . . . . . . . . . . . . . V . . . . S . . N . . . . . . . . . . V . . . . . .  450
Human-H2N2-AY210104.1.pro    . . . . . . . . . . A . . . . . . . . . D K P . . . . . . . . . . . . . . . . . . A . . . . . . . G  450
Swine-H1N1-KP404381.1.pro    S . . . . . . . . . . . . . . . . . . . . . T . . . . . . . . . . . . . . . . . . . . . . . . . . .  450
Chicken-H5N8-MF926454.1.pro  . . . . . . . . . . . . . . . . . . . . . . . . . . . . . . . . . . . . . . . . . . . . . . . . . .  450
Chicken-H7N7-KM593187.1.pro  . . . . . . . . . . . . . . . . . . . . . . . . . . . . . . . . . . . . . . . . . . . . . . . . . .  450
Chicken-H7N9-KM879323.1.pro  . . . . . V . . . . . . . . . . . . . . . . . . . . . . . . . . . . . . . . . . . . . . . . . . . . .  450
Chicken-H9N2-AF156411.1.pro  . . . . . . . . . . . . . . . . . . . . . . . . . . . . . . . . . . . . . . . . . . . . . . . . . .  450
Duck-H5N1-AY856864.1.pro     . . . . . . . . . . . . . . . . . . . . . . . . . . . . . . . . . . . . . . . . . . . . . G . . . .  450
Duck-H5N2-JX507356.1.pro     . . . . . . . . . . . . . . . . . . . . . . . . . . . . . . . . . . . . . . . . . . . . . . . . . .  450
Duck-H9N2-KP865772.1.pro     . . . . . V . . . . . . . . . . . . . . . . . . . . . . . . . . . . . . . . . . . . . . . . . . . . .  450
Canine-H3N2-MG254116.1.pro   . . . . . . . . . . . . . . . . . . . . . . . . V . . . . S . . N . . . . . . . . . . V . . . . . .  450
Canine-H5N2-HM765509.1.pro   . . . . . . . . . . . . . . . . . . . . . . . . . . . . . . . . . . . . . . . . . . . . . . . . . .  450

Majority                     A R P E D V S F Q G R G V F E L S D E K A T N P I V P S F D M S N E G S Y F F G D N A E E Y D N -
                                              460                 470                 480                 490

Equine-H3N8-EU794544.1.pro   A K S E D V S F Q G R G V F E L S D E K A T N P I V P S F D M S N E G S Y F F G D N A E E F D S .    499
Equine-H3N8-KF806988.1.pro   . . . . . . . . . . . . . . . . . . . . . . . . . . . . . . . . . . . . . . . . . . . . . . . . .    499
Equine-H3N8-M63786.1.pro     . R P . . A . . . . . . . . . . . . . . . . . . . . . . . . . . . . . . . . . . . . . . . Y . N .    499
Equine-H5N1-ON533586.1.pro   . R P . . . . . . . . . . . . . . . . . . . . . . . . . . . . N . . . . . . . . . . . . . Y . N .    499
Equine-H7N7-JX983551.1.pro   . R . . . . . . . . . . . . . . . . . . . . . . . . . . . . . . . . . . . . . . . . . . . . . . .    499
Human-H1N1-CY034135.1.pro    . . P . . L . . . . . . . . . . . . . . . . . . . . . . . . . . . . . . . . . . . . . . . Y . . .    499
Human-H1N1-GQ166225.1.pro    . . P . . L . . . . . . . . . . . . . . . . . . . . . . . . . . . . . . . . . . . . . . . Y . . .    499
Human-H2N2-AY210104.1.pro    . . P . E M . . . . . . . . . . . . . . . . . . . . . . . . . . . . . . . . . . . . . . . Y . N .    499
Swine-H1N1-KP404381.1.pro    . R P . . . . . . . . . . . . . . . . . . . . . . . . . . . . . . . . . . . . . . . . . . Y . N .    499
Chicken-H5N8-MF926454.1.pro  . R P . . . . . . . . . . . . . . . . . . . . . . . . . . . . . . . . . . . . . . . . . . Y . N .    499
Chicken-H7N7-KM593187.1.pro  . R P . . . . . . . . . . . . . . . . . . . . . . . . . . . . . . . . . . . . . . . . . . Y . N .    499
Chicken-H7N9-KM879323.1.pro  . R P . . . . . . . . . . . . . . . . . . . . . . . . . . . . N . . . . . . . . . . . . . Y . N .    499
Chicken-H9N2-AF156411.1.pro  . R P . . . . . . . . . . . . . . . . . . . . . . . . . . . . . K . . . . . . . . . . .              494
Duck-H5N1-AY856864.1.pro     . R P . . . . . . . . . . . . . . . . . . . . . . . . . . . . N . . . . . . . . . . . . . Y . N .    499
Duck-H5N2-JX507356.1.pro     . . P . . . . . . . . . . . . . . . . . . . . . . . . . . . . I . . . . . . . . . . . . . Y . N .    499
Duck-H9N2-KP865772.1.pro     . R P . . . . . . . . . . . . . . . . . . . . . . . . . . . . N . . . . . . . . . . . . . Y . N .    499
Canine-H3N2-MG254116.1.pro   . . P . . L . . . . . . . . . . . . . . . . . . . . . . . . . . . . . . . . . . . . . . . Y . . .    499
Canine-H5N2-HM765509.1.pro   . R P . A . . . . . . . . . . . . . . . . . . . . . . . . . . . . . . . . . . . . . . . . Y . N .    499
```

Decoration 'Decoration #1': Hide (as '.') residues that match Equine-H3N8-EU794544.1.pro exactly.
